# TMEM263: a novel candidate gene implicated in human autosomal recessive severe lethal skeletal dysplasia

**DOI:** 10.1186/s40246-021-00343-2

**Published:** 2021-07-08

**Authors:** Mahsa Sadat Asl Mohajeri, Atieh Eslahi, Zeinab Khazaii, Mohammad Reza Moradi, Reza Pazhoomand, Shima Farrokhi, Masoumeh Heidari Feizabadi, Farzaneh Alizadeh, Majid Mojarrad

**Affiliations:** 1 Department of Medical Genetics, Faculty of Medicine, Mashhad University of Medical Sciences, Mashhad, Iran; 2 Student Research Committee, Faculty of Medicine, Mashhad University of Medical Sciences, Mashhad, Iran; 3 Medical Genetics Research Center, Faculty of Medicine, Mashhad University of Medical Sciences, Mashhad, Iran; 4 Genetic Center of Khorasan Razavi, Mashhad, Iran; 5 Legal Medicine Research Center, Legal Medicine Organization of Iran, Tehran, Iran; 6 Genetic Department, Shiraz Fertility Center, Shiraz, Iran

**Keywords:** Skeletal dysplasia, Novel gene, Whole exome sequencing, Gene discovery

## Abstract

**Introduction:**

Skeletal dysplasia is a common, clinically and genetically heterogeneous disorder in the human population. An increasing number of different genes are being identified causing this disorder. We used whole exome sequencing (WES) for detection of skeletal dysplasia causing mutation in a fetus affected to severe lethal skeletal dysplasia.

**Patient:**

Fetus was assessed by ultrasonography in second trimester of pregnancy. He suffers from severe rhizomelic dysplasia and also pathologic shortening of ribs. WES was applied to finding of causal mutation. Furthermore, bioinformatics analysis was performed to predict mutation impact.

**Results:**

Whole exome sequencing (WES) identified a homozygous frameshift mutation in the TMEM263 gene in a fetus with severe lethal skeletal dysplasia. Mutations of this gene have been previously identified in dwarf chickens, but this is the first report of involvement of this gene in human skeletal dysplasia. This gene plays a key role in the growth hormone signaling pathway.

**Conclusion:**

TMEM263 can be considered as a new gene responsible for skeletal dysplasia. Given the complications observed in the affected fetus, the mutation of this gene appears to produce much more intense complications than that found in chickens and is likely to play a more important role in bone development in human.

## Introduction

Skeletal dysplasia is a common and heterogeneous developmental disorder, with more than 450 different disorders, associated with impaired longitudinal growth and bone mineralization. The disorder affects one in every 5000 live births, approximately [1].

Various environmental, teratogenic, and genetic factors are involved in causing this complication. Genetic factors play an important role in causing bone dysplasia and so far mutations in 226 different genes have been reported in patients with skeletal dysplasia [1].

Although the genetic cause of an increasing number of skeletal dysplasia syndromes is being discovered, the genetic etiology of many of these disorders remains unknown.

It is important to identify the genetic causes of skeletal dysplasia in genetic counseling, prenatal diagnosis, and prognosis of the patient.

The TMEM263 gene has recently been identified as one of the causes of autosomal recessive dwarfism in Cornell K-strain white leghorns [2]. Mutation of this gene results in short stature and weight loss despite normal levels of growth hormone and insulin-like factor 1 [3].

Interestingly, although no association has been observed with skeletal dysplasia so far, some GWAS studies have shown a significant association between TMEM263 genetic variants and bone mineral density (BMD) and bone fracture risk in humans [4].

In addition, bioinformatics studies of transcriptome data have shown concurrent expression of this gene and osteoblast functional modules (OFMs) [5].

In the present study, we identified for the first time the TMEM263 mutation in a fetus suffering bone skeletal dysplasia using WES technology and investigating autologous gene recognized in dwarf chicken.

## Patient and methods

### Clinical brief

**Fig. 1:**
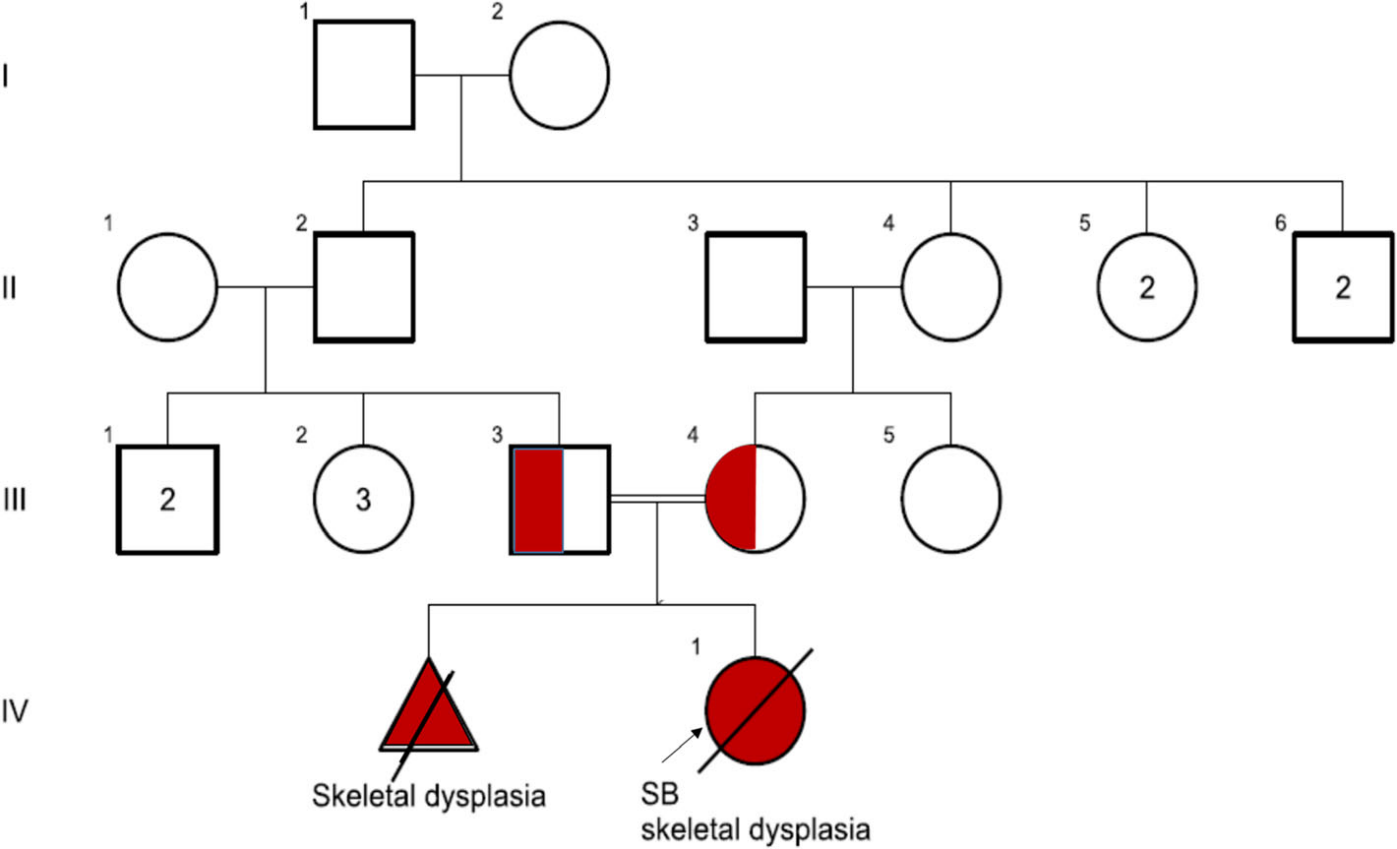
Family pedigree. Filled red symbols indicate members affected with skeletal dysplasia and half-filled shows heterozygote for the mutation and the proband is indicated with the arrow

A consanguineous couple with a fetus with fatal skeletal dysplasia was referred for genetic counseling before termination of pregnancy. In the initial study, couples had a previous pregnancy with the same problem (Fig. 1).

The couple underwent genetic counseling and ultrasound examination of the fetus. Amniocentesis was performed to culture of amniotic fluid cells.

The sample was a male aborted fetus. Fetal age at diagnosis was 15 week and 1 day based on first trimester indices, 16 weeks based on biparietal diameter )BPD(, and 16 weeks and 1 day based on abdominal circumference )AC( and the fetal weight was 85 g.

The fetal femur length was 6.5 mm (lower than 2.5th percentile of the normal population).

On ultrasonography examination, humerus length was 2 mm (lower than 5th percentile of the normal population) and tibial bone length was 1 mm (lower than 2th percentile of the normal population). Pathological shortening of ribs and other long bones was evident.

Furthermore, bilateral clubfoot and clubhand was apparent.

There was no evidence of cleft palate and lip. Gastric bubble, kidneys, and bladder appeared normal.

In color Doppler study, normal cord with two arteries and one vein was observed.

No abnormalities were observed in CNS examination and lateral ventricle diameter was within normal range.

Nuchal fold was in pathological range (5.7 mm) and nasal bone length was lower than 2.5th percentile of the normal population.

Doppler examination of fetal heart showed quadruple and triceps view and no pathologic findings.

Finally, according to these evidences, diagnosis of severe lethal micromelia was confirmed. Termination of pregnancy was performed under the supervision of a gynecologist.

In family history, similar findings were apparent in previous pregnancy. In addition, previous fetus shows several angulations suspected to fracture has been detected and interestingly, omphalocele has been detected in former fetus but not in later one.

### Whole exome sequencing and segregation analysis

After taking informed consent from parents, the WES assay was performed according to standard protocol. In brief, genomic DNA samples were extracted from cultured amniotic cells using DNA extraction kit (SIMBIOLAB, IRAN).

The whole exome captured using the Agilent SureSelect Target Enrichment Kit preparation guide. The libraries were sequenced with Illumina HiSeq 2000/2500 sequencer.

Reads were Mapped hg19 human reference genome assembly and cleaned to using BWA and Samtools, respectively [6, 7]. Duplicated reads were removed by using Samtools dedup function. Variant filtering was performed toward the SNPs and indels using VarScan v2.3.9, can generally run from 3 to 25 budgets [8]. Functional annotation for detected variants was performed using Wannovar [9].

Known skeletal dysplasia causing genes were extracted. Filtering of variants was done using multiple steps based on homozygosity status, allelic frequencies in different population databases, mutation impact on gene function, and mutation effect based on various prediction databases.

In next step, other preferred variants based on above algorithm were studied based on PubMed literature.

Sequence analysis was performed using the Sanger sequencing method to confirmation of TMEM263 candidate gene variant. Primers sequences are listed in Table 1.

**Table 1:** Primer sequence of TMEM263 mutation confirmation

Primer name	Sequence (5′ to 3′)	Tm	Amplification product length
TMEM263F	GAAAGATCACCCACAGCAG	52.63	237 bp
TMEM263R	TTTACAACAGCAGACCCAAC	45.00	

Polyphen, LIF, SIFT, MUTATION TASTER, PROVEAN softwares were used to predict the identified variant effects. The domain information of TMEM263 gene was obtained from pfam and interpro databases.

## Results

In the WES study, 123011 variant with an average depth of 50× and minimum depth of 8× was detected. 6254 SNPs and indels variants belong to known skeletal dysplasia causing genes were extracted, all of which were either intronic or common population polymorphisms greater than 2%.

In the second step, variant filtering was performed based on previously mentioned criteria.

Interestingly, a new variant was observed in the TMEM263 gene. This mutation is located in exon 4 of the gene, resulting in the deletion of 188-189th nucleotides, resulting in a shift of the reading frame from amino acid lysine number 63 onwards resulting in the creation of a premature stop codon in the 68th residue.

This variant was not reported in the 1000G, ExAC, gnomAD, and also Iranome. Furthermore, this mutation was not observed in our local database, which contained 400 WES data from the eastern part of Iran. So, this variant is a family specific mutation which may lead to this phenotype.

Polyphen, LIF, SIFT, MUTATION TASTER, PROVEAN softwares detect the identified mutation as either deleterious or possibly damaging or pathogenic.

Examination of the protein sequence from the mutation revealed the elimination of the majority of the second trans-membrane domain of protein which by eliminating that the function of this protein is virtually lost.

**Fig. 2:**
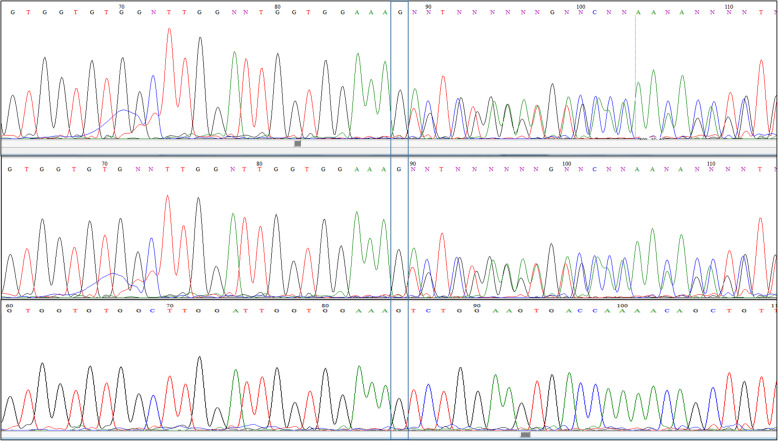
Partial sequence chromatogram displaying the DNA sequence of parents. The arrowhead indicates the position of the homozygous one-nucleotide deletion (c.188_189delAG) resulting in a frameshift mutation

In addition, this mutation may also result to nonsense mediated mRNA decay (NMD), which would completely silence TMEM263 protein expression. Sequence analysis showed heterozygous status in both parents (Fig. 2).

## Discussion

In this study, using WES, we have identified TMEM263 mutation as a new severe lethal skeletal dysplasia causative mutation. This gene is located on chromosome 12q23.3 and contains 4 exons, which only exons 3 and 4 are encoding, producing a protein of 116 amino acids in length.

Few studies have been performed on TMEM263 gene and the physiological role of this gene remains largely unknown. However, the study of Wu et al. has shown that TMEM263 protein is highly conserved among all vertebrates [2]. This protection is particularly apparent in the two trans-membrane domains (units 38 to 61 equivalent to the first domain and 80 to 102 units to the second domain). In human TMEM263 protein, the two domains comprise the first domain 40-60 and the second domain subunit 98-78.

TMEM263 is expressed in a wide range of tissues at different stages of development and has the highest expression level in tibia. Interestingly, the expression of this gene in tibia in the samples of individuals 6 to 12 years is higher than in adults.

A nonsense mutation detected in the Trp59 residue in the dwarf Cornell K-strain white leghorns results in the deletion of the second domain of the protein, resulting in the protein completely losing its function. Interestingly, our mutation occurred in Lysine 63, leading to the early termination of the protein [2].

TMEM263 directly interacts with GH1 and BMP2, both of which are essential for the normal growth and development of long bones [2]. In addition, based on the data obtained from GeneMANIA, the protein TMEM263 also coexpressed with FAM3C and RBPJ proteins. These genes have known effects on osteogenesis and bone density in humans [10–12].

Recent GWAS studies also show a significant association of TMEM263 variants with bone mineral density in humans [4, 13].

On the other hand, co-immunopercipitation studies on the Slick protein, a sodium-activated potassium channel, indicate physical binding of this protein to TMEM263 [14]. Because dysfunction of the Slick gene leads to infantile epileptic encephalopathy type 57, TMEM263 gene dysfunction may also be involved in genetic disorders of the nervous system and epilepsy syndromes [15]. However, the lack of neurological effects in the animal model of this mutation suggests that this gene may not play a critical role in the central nervous system.

Another important point is that the consequences of this mutation in the human fetus appear to be much more severe than the complications of the chicken, suggesting a higher physiological importance of this gene in humans than in the chicken.

## Conclusion

In this study, we identified TMEM262 as a novel gene involving in skeletal dysplasia. This finding can be used to screening of patients with skeletal dysplasia.

## Data Availability

Mentioned in the text.
